# Transmission of Staphylococcus aureus from Humans to Green Monkeys in The Gambia as Revealed by Whole-Genome Sequencing

**DOI:** 10.1128/AEM.01496-16

**Published:** 2016-09-16

**Authors:** Madikay Senghore, Sion C. Bayliss, Brenda A. Kwambana-Adams, Ebenezer Foster-Nyarko, Jainaba Manneh, Michel Dione, Henry Badji, Chinelo Ebruke, Emma L. Doughty, Harry A. Thorpe, Anna J. Jasinska, Christopher A. Schmitt, Jennifer D. Cramer, Trudy R. Turner, George Weinstock, Nelson B. Freimer, Mark J. Pallen, Edward J. Feil, Martin Antonio

**Affiliations:** a Medical Research Council Unit, Fajara, The Gambia; b University of Warwick, Coventry, United Kingdom; c University of Bath, Bath, United Kingdom; d University of California Los Angeles, Los Angeles, California, USA; e American Military University and American Public University, Charles Town, West Virginia, USA; f University of Wisconsin—Milwaukee, Milwaukee, Wisconsin, USA; g University of the Free State, Bloemfontein, South Africa; h Washington University, St. Louis, Missouri, USA; i London School of Hygiene and Tropical Medicine, London, United Kingdom; University of Helsinki

## Abstract

Staphylococcus aureus is an important pathogen of humans and animals. We genome sequenced 90 S. aureus isolates from The Gambia: 46 isolates from invasive disease in humans, 13 human carriage isolates, and 31 monkey carriage isolates. We inferred multiple anthroponotic transmissions of S. aureus from humans to green monkeys (Chlorocebus sabaeus) in The Gambia over different time scales. We report a novel monkey-associated clade of S. aureus that emerged from a human-to-monkey switch estimated to have occurred 2,700 years ago. Adaptation of this lineage to the monkey host is accompanied by the loss of phage-carrying genes that are known to play an important role in human colonization. We also report recent anthroponotic transmission of the well-characterized human lineages sequence type 6 (ST6) and ST15 to monkeys, probably because of steadily increasing encroachment of humans into the monkeys' habitat. Although we have found no evidence of transmission of S. aureus from monkeys to humans, as the two species come into ever-closer contact, there might be an increased risk of additional interspecies exchanges of potential pathogens.

**IMPORTANCE** The population structures of Staphylococcus aureus in humans and monkeys in sub-Saharan Africa have been previously described using multilocus sequence typing (MLST). However, these data lack the power to accurately infer details regarding the origin and maintenance of new adaptive lineages. Here, we describe the use of whole-genome sequencing to detect transmission of S. aureus between humans and nonhuman primates and to document the genetic changes accompanying host adaptation. We note that human-to-monkey switches tend to be more common than the reverse and that a novel monkey-associated clade is likely to have emerged from such a switch approximately 2,700 years ago. Moreover, analysis of the accessory genome provides important clues as to the genetic changes underpinning host adaptation and, in particular, shows that human-to-monkey switches tend to be associated with the loss of genes known to confer adaptation to the human host.

## INTRODUCTION

Staphylococcus aureus is an important pathogen of humans, causing a range of conditions from serious invasive diseases such as meningitis, pneumonia, and bacteremia to less severe skin and soft tissue infections (1). S. aureus is among the top five most common causes of bacteremia in sub-Saharan Africa and the second leading cause of bacteremia in The Gambia (2–4). S. aureus thus poses a serious public health burden in The Gambia, yet little is known about the population structure and dynamics of this pathogen in sub-Saharan Africa. In other parts of the world, interest has focused on the role of nonhuman hosts (mostly livestock) as reservoirs of infection and drug resistance relevant to humans (5–7). In addition, it is clear that S. aureus can switch host species, sometimes resulting in adaptation to the new host and onward transmission in the new host species (8).

Interspecies transmission and adaptive host switching are known to occur between humans and nonhuman primates. Human-associated S. aureus lineages readily colonize and infect nonhuman primates in captivity and in the wild (9–12). In remote regions of Africa, wild monkeys are mainly colonized by S. aureus isolates belonging to uncharacterized clonal complexes that rarely colonize or infect humans, with one highly divergent clade isolated from monkeys in sub-Saharan Africa now classified as a new species, Staphylococcus schweitzeri (13, 14).

The gain or loss of genes associated with mobile genetic elements is thought to be the primary driver of host adaptation following interhost transmission (15). Nonhuman hosts provide an environment for the acquisition of novel virulence and resistance determinants (16). For example, clones of methicillin-resistant Staphylococcus aureus (MRSA) from human-associated lineages such as clonal complex 5 (CC5), CC9 and sequence type 88 (ST88) have been reported in livestock (17) and livestock-associated MRSA lineages, most notably CC97 and CC398, have overcome the species barrier to infect humans (15, 18).

In The Gambia, increasing urbanization and tourism have meant that wild green monkeys have become habituated to humans, resulting in increased opportunities for interhost transmission of potential pathogens. In particular, free-ranging wild monkeys inhabit the Bijilo Forest Park which is close to the Senegambia tourist area and serves as a tourist attraction where locals and tourists go to visit the monkeys. Although feeding the animals is prohibited, people take bags of peanuts into the park and feed the monkeys by hand. Here, we describe the use of whole-genome sequencing to detect transmission of S. aureus between humans and nonhuman primates and to document the genetic changes accompanying host adaptation.

## MATERIALS AND METHODS

### Study isolates.

We pooled isolates from three previous studies that characterized S. aureus from monkeys, human carriage, and invasive disease by multilocus sequence typing (MLST) and antimicrobial susceptibility testing (19, 20). The first study was conducted in 2011 by the International Vervet Research Consortium on simian immunodeficiency virus (SIV) infection in green monkeys (Chlorocebus sabaeus) in The Gambia (20). Thus, we were able to collect nasopharyngeal swabs (NPS) and oropharyngeal swabs (OPS) from the monkeys. Eighty-two S. aureus isolates were cultured from 64 NPS and 63 OPS collected from 64 human-habituated wild monkeys in The Gambia. In the second study, 100 S. aureus strains were isolated from human NPS as part of a carriage study conducted between December 2005 and April 2005 in Sibanor (19). The third study analyzed a selection of 116 S. aureus strains isolated from archived clinical specimens of patients who reported to the Medical Research Council (MRC) clinic in Fajara with invasive bacterial disease between 2002 and 2010 (unpublished data). Table S1 and Fig. S4 in the supplemental material show the temporal spread of sampling in the different epidemiological classes and the spatial distribution of the sites where the samples were collected.

To isolate S. aureus, specimens were plated on mannitol salt agar (MSA) (Oxoid, Basingstoke, United Kingdom) and 5% sheep blood agar (BA) (Oxoid, Basingstoke, United Kingdom) plates. The specimens were incubated at 37°C for 24 h on BA plates and 48 h on MSA plates under aerobic conditions. Suspected S. aureus colonies were subcultured on BA plates for 24 h and confirmed by a coagulase test using the Slidex Staph kit (bioMérieux, Basingstoke, Hampshire, United Kingdom). Antimicrobial susceptibility testing was performed by the disk diffusion method on BA plates for the following antibiotics: penicillin, co-trimoxazole, tetracycline, chloramphenicol, gentamicin, cloxacillin, erythromycin, and cefoxitin (Oxoid, Basingstoke, United Kingdom). Results were interpreted according to the guidelines of the Clinical and Laboratory Standards Institute (CLSI) (21).

### DNA extraction and MLST analysis.

Genomic DNA was extracted from fresh overnight pure cultures of S. aureus strains using the Qiagen genomic DNA extraction kit (Qiagen, United Kingdom) according to the manufacturer's protocol. MLST was performed on S. aureus isolates targeting seven housekeeping genes (*aroE*, *ptA*, *glp*, *arcC*, *gmK*, *tpi*, and *yqiL*) as described previously (22). An eBURST (23) analysis was performed on all STs of Staphylococcus aureus in the MLST database (http://saureus.mlst.net). STs were assigned to clonal complexes where they had 6 identical alleles with at least one other ST within the clonal complex. eBURST groups sequence types based on shared alleles, but it does not take into consideration the existing knowledge of the clonal population structure of S. aureus. As a result, in some instances, eBURST merged two or more clonal complexes that are well described in the literature into one eBURST group. In these cases, the clonal complex designations from the literature were used instead of the eBURST grouping in order to maintain consistency with the literature.

### Whole-genome sequencing, assembly, and annotation.

Ninety isolates were analyzed for whole-genome sequencing: 46 isolates from invasive disease in humans, 13 human carriage isolates, and 31 monkey carriage isolates. These isolates included at least one representative of all the major clonal complexes inferred from MLST. Whole-genome sequencing was carried out on the Illumina MiSeq system with the Nextera X preparation kit. *De novo* contigs were generated for each genome using SPAdes (kmers 21, 33, 55, 77, 99, and 127) (24). Contigs shorter than 300 bp and with kmer coverage of <2 were removed from the assemblies. Coding sequences (CDSs) were predicted and annotated by Prokka (version 1.11) (25).

### MLST from the whole genome.

To determine whether any isolates might have been misassigned in the MLST or whole-genome sequencing workflows, we used the draft genomes to predict multilocus sequence types. Two methods were employed: an assembly-based approach and a mapping-based approach. Assemblies were subjected to BLAST searches against all of the alleles in the MLST database (http://saureus.mlst.net). Alleles were called whenever there was 100% sequence coverage and 100% nucleotide identity. The mapping approach used SRST2 to map the reads to the seven housekeeping loci and identify the ST (26). The two sets of results were combined and manually curated. STs presented in Table S1 in the supplemental material are STs inferred from the whole genome that share at least 5 identical loci with the MLST results from the laboratory and belong to the same clonal complex. Eleven isolates were excluded from further analysis because the ST derived from the conventional MLST analysis differed from the ST inferred from the whole genome by more than two loci, leading us to believe that isolates had been misassigned in one analysis or the other.

### Phylogenetic analysis.

Sequencing reads were mapped to the EMRSA15 HO 5096 0412 reference genome (accession number HE681097) using SMALT (http://www.sanger.ac.uk/resources/software/smalt/). Single nucleotide polymorphisms (SNPs) were called using SAMtools 0.1.18, the Genome Analysis Toolkit (GATK), and in-house scripts (27, 28). SNPs were called from the core genome after exclusion of known repeat regions, insertion sequences, and known horizontally acquired elements. An approximate maximum likelihood phylogenetic tree was reconstructed using FastTree (29). Where appropriate, we included a reference genome belonging to each CC in the phylogenetic analysis. Isolates of Staphylococcus argenteus and Staphylococcus schweitzeri were included to ensure correct species identification (14).

### Accessory genome analysis.

Representative core and accessory genomes for the data set were identified using Roary on default settings (30). A pairwise matrix was generated, showing the proportion of shared accessory CDSs (see Fig. 2). Finding the set differences between clade 2 and the remaining isolates identified the accessory genome content specific to clade 2. CDSs found in more than 20 of the 22 clade 2 isolates were considered to potentially contribute to monkey-specific host adaptation.

The presence or absence of a number of genes associated with virulence in S. aureus was inferred by a BLAST search of the assembled genomes. Nucleotide sequences for virulence-associated genes alpha-hemolysin (*hlA*), beta-hemolysin (*hlB*), delta-hemolysin (*hlD*), staphylococcal enterotoxins A (*seA*), B (*seB*), C (*seC*), G (*seG*), H (*seH*), and I (*seI*), toxic shock syndrome toxin gene 1 (*tst1*), and Panton-Valentine leukocidin (PVL) genes (*lukF-PV* and *lukS-PV*) were identified from the Virulence Factors of Pathogenic Bacteria database (http://www.mgc.ac.cn/VFs/). The presence of the virulence genes in isolates used in this study was identified by using BLAST against the whole-genome assemblies with a cutoff of >90% base identity and length similarity to the reference gene.

### Genomic divergence.

To visualize genetic divergence across the genomes in clade 2, monkey isolates H7, F2, G2, G11, F7, and H10 were compared the finished closed genome USA300 FPR3757 using BLAST Ring Image Generator (BRIG) (31). Query genomes represented subclusters within the monkey-associated clade 2. USA300 FPR3757 was selected for comparison because of the presence of νSaα and νSaβ and the high quality of its annotation. The lower bounds of nucleotide similarity were set to 85%. Well-characterized mobile genetic elements were annotated for comparison.

### Ethical approval.

The Gambia Government/MRC Joint Ethics Committee approved the carriage study and the sampling of biological samples from the green monkeys. The Gambia Government/MRC Joint Ethics Committee gave subsequent approval to send genomic DNA of 96 S. aureus isolates to the University of Warwick, United Kingdom, for whole-genome sequencing.

### Accession number(s).

The genome sequence data from this study have been uploaded to the European Nucleotide Archive under the study accession number PRJEB12419.

## RESULTS

### Genomic analysis.

We analyzed 90 genomes of Staphylococcus aureus, representing 46 isolates from human invasive disease, 13 human carriage isolates, and 31 monkey pharyngeal isolates (see Table S1 in the supplemental material). Draft assemblies ranged in size from 2.7 to 3.4 Mb with a range of 2,462 to 2,921 coding sequences. When mapped to the EMRSA-15 reference genome, the mean average coverage was 61.5-fold (range, 12.5- to 126.2-fold).

### Phylogenetic analysis.

**FIG 1:**
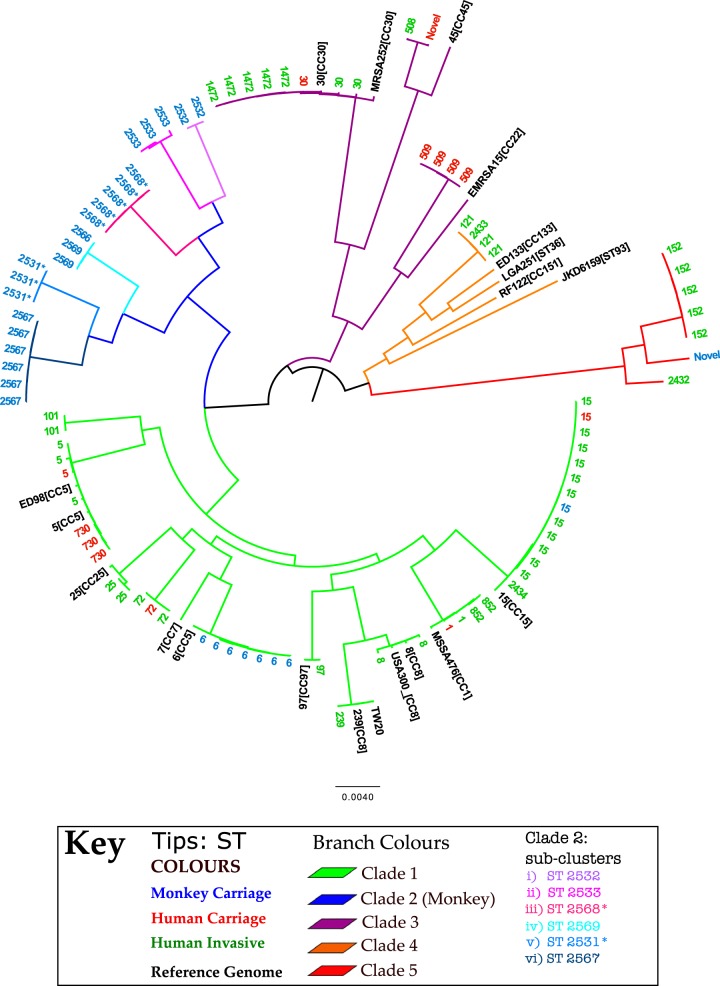
A maximum likelihood phylogenetic tree showing 5 major clades; branches are colored based on clade assignment. Tips are annotated by ST and colored by host. Reference genomes are annotated by name and CC and colored black. *, single locus variant of given ST.

Single nucleotide polymorphisms (SNPs) were called from 232,276 sites in the core genome after exclusion of all known repeat regions and transposable mobile elements. We created a maximum-likelihood phylogenetic tree linking our isolates with reference strains. This tree resolved five major S. aureus clades that are consistent with previous species-wide phylogenetic analyses (Fig. 1; see also Fig. S2 in the supplemental material). Clade 1 encompasses the well-described CCs 8, 1, 5, 15, and 25. Clade 2 is a novel monkey-associated clade encompassing six subclusters. Clade 3 encompasses CC30 and CC45. Clade 4 corresponds to CC121 (22) and various animal-associated reference genomes. Finally, clade 5 corresponds to ST152 and related genotypes.

Current estimates of intraclonal mutation rates are consistent in S. aureus (see the supplemental material in reference 32). Based on the intraclonal mutation rate for S. aureus of ∼2 × 10^−6^ per site per year (32) and a core genome size of ∼2.5 Mb, we were able to estimate an upper bound date for the transmission events. The short-term mutation rate equates to about 5 SNPs per year. Thus, simply dividing the total number of SNPs between any pair of contemporary S. aureus isolates by 10 gives the approximate number of years since they shared a common ancestor.

The single monkey-derived ST15 isolate differs by only 71 core genome SNPs from the most closely related human-derived ST15. Assuming equal rates of mutation in both lineages, this represents approximately 7 years divergence. However, in this case, the estimated time of interhost transmission is likely to represent a misleadingly high upper bound, as we are very unlikely to have sampled the human-associated relative closest to the transmitted strain. Furthermore, the fact that ST15 was only found once in monkeys and that there is no evidence of onward transmission is consistent with a very recent transmission event.

We identified a cluster of seven ST6 isolates derived from monkeys. An interrogation of the MLST database (www.mlst.net) revealed that this genotype is occasionally recovered from cases of bovine mastitis but is predominantly human associated. Therefore, the cluster of seven ST6 isolates recovered from monkeys is also likely to represent recent anthroponotic transmission. The closest pairwise SNP distance between a monkey ST6 isolate and the ST6 reference strain was 311 SNPs, suggesting that the host switch happened no more than 3 decades ago. The most divergent pair of monkey-derived ST6 isolates differed by 270 SNPs, which also equates to around 3 decades of divergence. However, if this cluster represents multiple transmission events from humans to monkeys, then the resulting host switches might have occurred more recently.

The third host switching event evident from our analysis is the much more ancient event that gave rise to the diverse monkey-associated clade 2, which was characterized by isolates with novel STs not found in humans. As this clade is nested within predominantly human-associated lineages (Fig. 1), it is likely to have resulted from an ancient human-to-monkey transmission event. The smallest SNP difference between a clade 2 isolate and a non-clade 2 isolate (in this case the reference MSSA476) is 26,968 SNPs, which equates to ∼2,700 years of divergence. This may be an underestimate, since our rate estimate is based on divergence over much shorter time scales (which is likely to be more rapid due to the lag time of purifying selection).

### Accessory genome variation and host adaptation.

**FIG 2:**
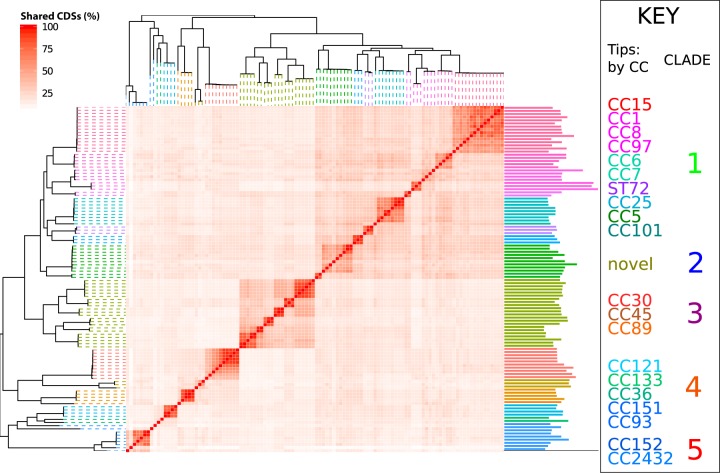
A heat map nested into the phylogeny showing the proportion of accessory genes that are shared between isolates, determined by a pairwise comparison. Branches on the phylogeny are colored based on the clonal complex. Darker squares in the heat map indicate higher shared accessory genome content. To the right of the heat map is a bar chart showing the number of accessory genes for each genome.

Genomes corresponding to the same clonal complex also share similar accessory gene content (Fig. 2). Moving out to a broader phylogenetic scale, related CCs share more similar accessory gene content than unrelated CCs (e.g., the large square corresponding to the CC15/1/8/97/6/25/5 clade). This confirms previous observations using microarray data, which led to the concept of the “core variable” genome, meaning those genes that are stably present or absent within a given clonal complex but vary in their distribution between clonal complexes (33).

**FIG 3:**
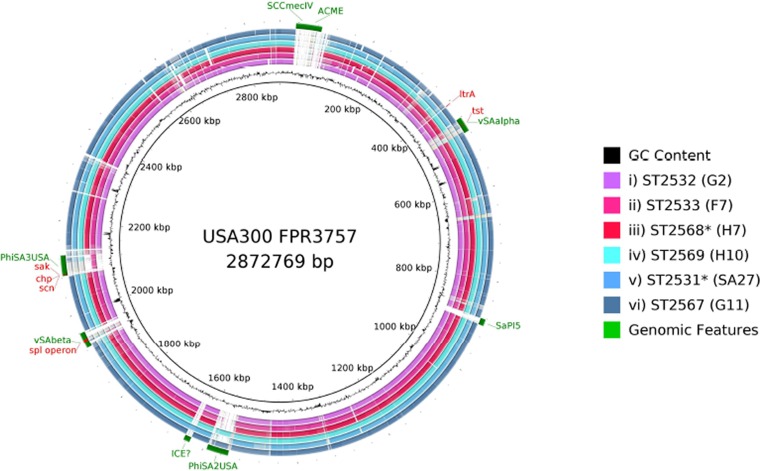
BRIG. Whole-genome sequence analysis and comparison of USA300_FPR3757 to isolate a representative from each of the six subclusters in the monkey-associated clade 2. Circular diagram of the USA300 chromosome showing (from inner to outer), percent G+C, GC skew, and the homology based on BLASTN analysis of USA300_FPR3757 to H7, F2, G2, G11, F7, and H10. The sequence similarity ranges from 85% to 100%, as regions of homology of <85% were excluded. The outermost circle shows the location of large horizontally acquired pathogenicity islands and well-characterized phages present in USA300. *, single locus variant of given ST.

Variations in accessory gene contents were observed within the different clade 2 subgroups (Fig. 3). Regions of difference mostly represented horizontally acquired elements such as the genomic islands νSaα and νSaβ, *SCCmec*, and phage and pathogenicity islands. Genes present in νSaα but absent or in low frequency within clade 2 include a variant of *tst1* (toxic shock syndrome precursor) and genes encoding two superantigen-like proteins, two putative leukocidins, and a 65-kDa membrane protein. The *ltrA* gene was present in all strains except those in clade 2 and the CC152 strains of clade 5 (see the supplemental material). This gene encodes a “low temperature requirement” protein (Pfam: PF06772.5, COG4292) found to be essential for growth at low temperatures (4°C) in Listeria monocytogenes (34), but its function in S. aureus is unknown.

The *spl* operon resides in the νSaβ pathogenicity island and encodes extracellular serine proteases. The distribution of genes within this operon is consistent with a role in host adaptation. The variants of the *splA* to *splE* genes present in the USA300_FPR3757 reference are missing in all clade 2 isolates. However, clade 2 isolates contain novel variants of *spl* genes that are missing in all other isolates.

The immune evasion cluster (IEC1) proteins *sak*, *scn*, and *chp*, which are harbored on the phage φSA3 (Fig. 3), are absent from clade 2. All three genes are absent in the ST6 cluster, with the exception of *sak* and *scn* present in isolates E3 and E8. Furthermore, *sak* and *scn* are absent in SA29 (the ST15 monkey-derived isolate), and all three genes are absent in the monkey-derived CC152 isolate F5. This last example is particularly notable as the human-associated CC152 isolates in our collection contain these genes (see Data Set S1 and Fig. S1 in the supplemental material). The IEC1 genes are known to be associated only with human isolates and thus are thought to be involved in host-specific functions (35–37). Our analysis also confirms the absence of these genes in the animal-derived reference genomes ED98 (chicken), LG251 (cow), ED133 (sheep), and RF122 (cow) (35).

The *lytN* gene encodes a murein hydrolase thought to contribute to the release of protein A (a major immunoglobulin binding protein) from the cell surface by the removal of sugars (38). We note two variants of this gene in our data. Variant lytN is present in 18/22 clade 2 isolates but in only 10/88 non-clade 2 isolates (4 of which are from the monkey-associated ST6 cluster). Variant lytN_2 is absent from all 22 clade 2 isolates but present in 68/88 of all other isolates.

The Panton-Valentine leukocidin (PVL) genes (*lukF-PV* and *lukS-PV*) were absent from all monkey isolates in the data set. This observation supports the adaptation to the new host since it works in humans but not monkeys (36). The staphylococcal enterotoxins A (*seA*), B (*seB*), C (*seC*), G (*seG*), H (*seH*), and I (*seI*), were absent from all monkey isolates. The exception was the presence of enterotoxin A (*seA*) in two ST6 monkey isolates. Beta-hemolysin (*hlB*) was absent from all human isolates except the ST152 human isolates.

### Antibiotic resistance profiles.

We found no evidence of methicillin-resistant S. aureus (MRSA) among our staphylococcal isolates from monkeys in The Gambia. One monkey isolate (G9) was reported as methicillin resistant by the cefoxitin disc diffusion test, but the Etest confirmed that it was susceptible to methicillin. In addition, no genomic evidence of methicillin resistance was found when the genome was analyzed using Mykrobe (39) Two invasive disease isolates were confirmed to be MRSA through phenotypic testing; the *mecA* gene was present in both isolates. To our knowledge this is the first report of MRSA causing human invasive disease in The Gambia.

## DISCUSSION

Using whole-genome sequencing, we infer multiple human-to-monkey transmission events, but no evidence of monkey-to-human transmission. This observation is consistent with the report by Schaumburg et al., who used MLST and *spa* typing to compare human and monkey staphylococcal isolates from three African countries, Côte d'Ivoire, Gabon, and Democratic Republic of Congo. Their findings revealed numerous examples of human-to-monkey transmission but no evidence of the reverse (13).

A consistent picture of a clonal population structure, in which closely related strains cluster into several widespread clonal complexes (CCs) that are clearly delineated from each other, has emerged from our data. The majority of S. aureus colonizing in monkeys was due to novel lineages that formed clade 2 in the phylogenetic tree (Fig. 1). The phylogenetic placement of clade 2 suggests that it arose from an ancient human-to-monkey transmission event. This clade is believed to have diverged from human S. aureus ∼2,700 years ago, long before modern human population expansion and its ecological consequences. Over time, this clade appears to have adapted to the monkey host and has undergone clonal expansion.

The more recent transmission of human-associated lineages ST15 and ST6 to monkeys is believed to be a product of human encroachment into the natural habitat of monkeys and probably a result of transfer of bacteria from human hands to food, which is then fed to the monkeys. These two STs have been previously detected in African monkeys from remote regions of sub-Saharan Africa (13).

Analysis of the distribution of accessory genes between the monkey- and human-associated isolates confirmed the previous suggestion that genes carried on mobile genetic elements play a key role in host adaptation (40). Of particular note is the roles of the two genomic islands νSaα and νSaβ that encode superantigens, lipoproteins, and proteases. Gene contents within these islands differ markedly between strains as these islands recombine at high rates and are transferable by transducing phage particles (40), so that they have been referred to as “enterotoxin nurseries” (41, 42).

Gene loss may be as important as gene acquisition in the context of host adaptation. For example, isolates recovered from nonhuman hosts often harbor truncated variants of surface proteins present within closely related human isolates (43, 44). The phage-borne genes *chp*, *sak*, and *scn* that constitute the immune evasion cluster IEC1 have been previously noted to be exclusively associated with human-associated isolates, and our data are consistent with this view. Assuming that gene loss is an evolutionarily more parsimonious event than gene gain, this striking association may help to explain why S. aureus anthroponoses are more common than zoonosis, although we note not all human isolates harbor these genes (for example, they are absent within CC15). The association between host and variants of the *spl* operon is intriguing since it encodes serine proteases, as well as genes (*lytN*) involved in the processing of the major surface antigen staphylococcal protein A.

Reassuringly, we find no evidence of transmission of S. aureus from monkeys to humans. An analysis of MLST data has shown that for S. aureus there are generally higher rates of anthroponoses (human-to-animal transmission, *n =* 13) than zoonosis (animal-to-human transmission, *n =* 2) (8).

### Limitations of this study.

In this study, we did not perform *de novo* sample collection over a standard harmonized time frame but instead made use of existing sets of isolates collected at various times and in various places within The Gambia. Therefore, it seems unlikely that the humans in closest contact with the monkeys will have been sampled as part of this study. This has led us to temper our conclusions on the frequency of and direction of transmission, which could only be established in detail by a longitudinal study.

## Supplementary Material

Supplemental material
