# Supplementary material for: Transmission of Staphylococcus aureus from Humans to Green Monkeys in The Gambia as Revealed by Whole-Genome Sequencing
Source: Appl Environ Microbiol. 2016 Sep 16;82(19):5910–7. doi: 10.1128/AEM.01496-16 (PMC5038045; doi:10.1128/AEM.01496-16)
Supplement: Supplemental material [file supp_82_19_5910__index.html]

Supplemental material 

# Transmission of Staphylococcus aureus from Humans to Green Monkeys in The Gambia as Revealed by Whole-Genome Sequencing

## Supplemental material

- Supplemental file 1 -

  A heat map showing the presence and absence of accessory genes related to host adaptation across all genomes (Fig. S1), neighbor-joining tree (Fig. S2), phylogenetic tree linked to a heat map (Fig. S3), summary of sampling information for three epidemiological classes in the study (Table S1), and a map indicating the sites for the isolates (Fig. S4).

  PDF, 462K
- Supplemental file 2 -

  Isolate metadata (Data Set S1).

  XLSX, 14K
- Supplemental file 3 -

  Pairwise distance matrix showing the numbers of SNP differences within the core genome of isolates (Data Set S2).

  XLSX, 124K
- Supplemental file 4 -

  Presence (based on >85% homology) or absence of all accessory genes in all genomes (each row representing one gene and each column representing one isolate) (Data Set S3).

  XLSX, 3.4M
- Supplemental file 5 -

  A pairwise matrix showing the numbers of shared accessory genomes between all genomes (shared content based on the presence of two genes with at least 85% sequence similarity in both isolates) (Data Set S4).

  XLSX, 101K
