# Supplementary material for: Transmission of Staphylococcus aureus from Humans to Green Monkeys in The Gambia as Revealed by Whole-Genome Sequencing
Source: Appl Environ Microbiol. 2016 Sep 16;82(19):5910–7. doi: 10.1128/AEM.01496-16 (PMC5038045; doi:10.1128/AEM.01496-16)
Supplement: Supplemental material [file AEM.01496-16_zam999117421so1.pdf]

Supplementary Figure 1: A heatmap showing the presence and absence of accessory genes related to host adaptation across all genomes.

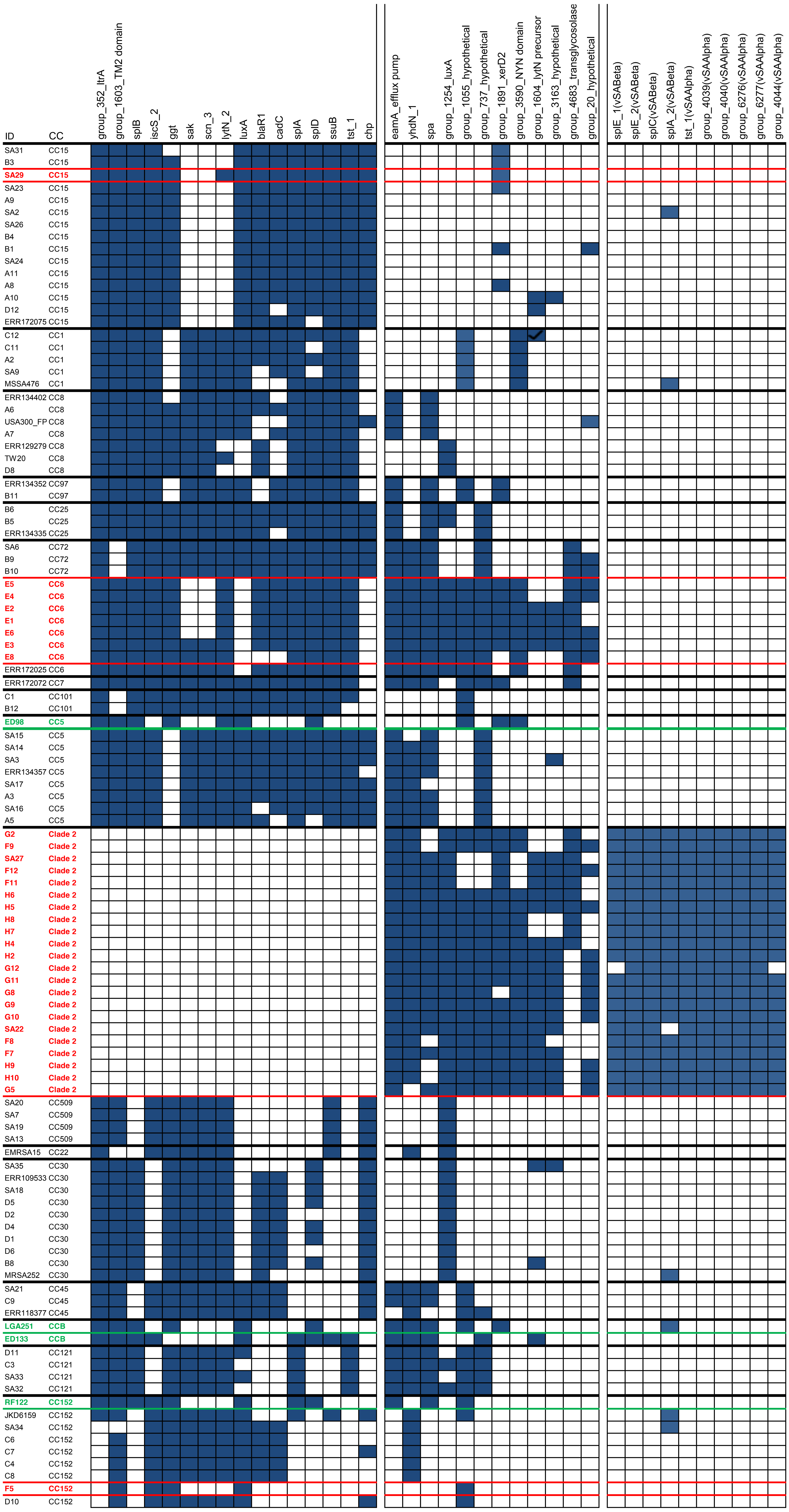

Monkey-derived isolates are highlighted in red and reference genomes are highlighted in green. Navy-blue circles indicate presence of the gene or variant of the gene in the isolate. The sequence similarity ranges from 85% to 100 %, as regions of homology <85% were excluded.

Supplementary figure 2 A neighbour joining tree including Staphylococcus schweitzeri and Staphylococcus argentius isolates. Tree shows the clade 2 isolates cluster clearly with other Staphylococcus aureus in the dataset. The phylogenetic placement of well characterised S. aureus genomes with our isolates is evidence that our isolates are S. aureus species.

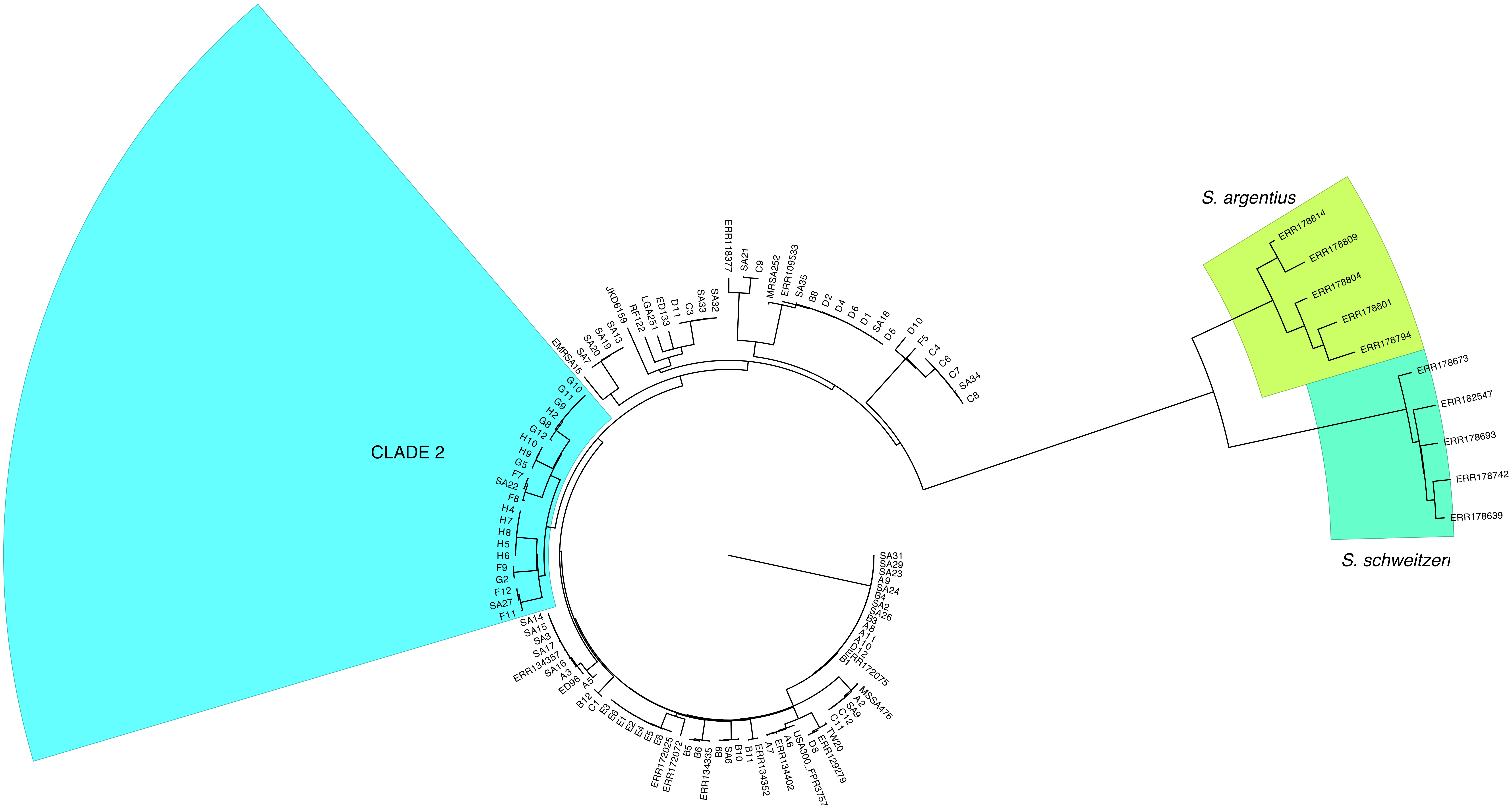

Staphylococcus schweitzeri and Staphylococcus argentius isolates from Tong et al., 2015 were mapped against EMRSA15 using the methods previously described (ENA accession numbers: S. argentius: ERS140248, ERS140026, ERS140254, ERS140095, ERS154949. S. schweitzeri ERS140147, ERS140266, ERS140239, ERS140159, ERS140162 and ERS140167). A neighbour joining tree with the S.aureus, S. argentius and S. schweitzeri was built using rapidNJ.

0.02

Supplementary figure 3 virulence figure. Phylogenetic tree linked to heatmap showing presence and absence of known selected enterotoxins.

Note: beta-haemolysin was present in almost all isolates in a truncated form (~82% of the gene). Alpha-haemolysin in F12 was truncated.

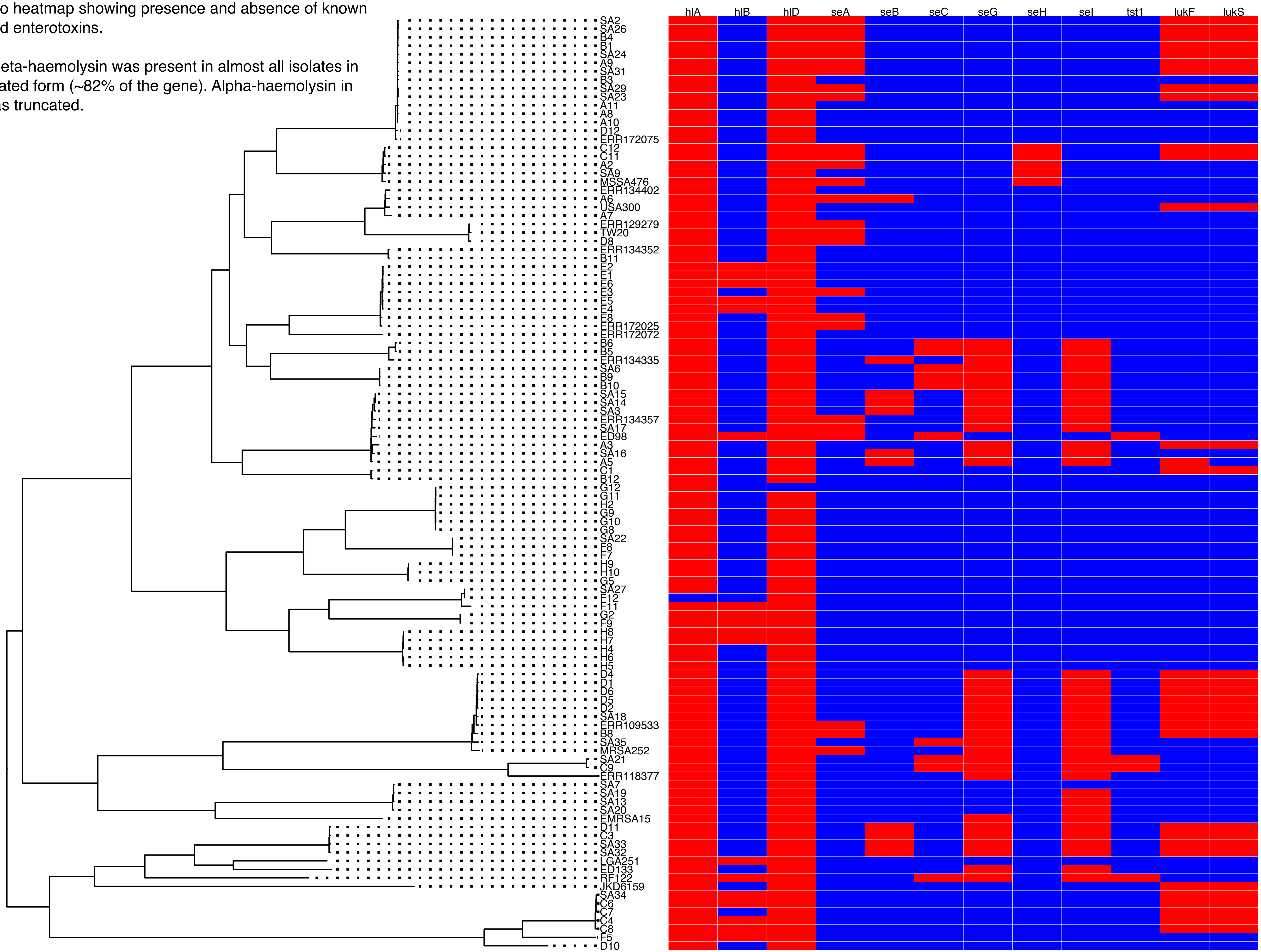

Nucleotide sequences for virulence associated genes alpha-hemolysin (hlA), beta-hemolysin (hlB), delta-hemolysin(hlD), staphylococcal enterotoxins A (seA), B (seB), C (seC), G (seG), H (seH) and I (seI), toxic shock syndrome toxin gene 1 (tst1) and Panton-Valentine leukocidin genes (lukF-PV and lukS-PV) were identified from the Virulence Factors of Pathogenic Bacteria database (http://www.mgc.ac.cn/Vfs/). The presence of the virulence genes in isolates used in this study was identified using BLAST against the whole genome assemblies with a cutoff of >90% base identity and length similarity to the reference gene. Red = presence and Blue = absence.

Supplementary table 1. Table showing a summary of the sampling information for three epidemiological classes in our study including approximate dates of isolation and geographical data.

Epidemiology	Study site	Start date	End Date	MLST analysis done	Whole Genome Analysis
Human invasive disease	MRC Clinic Fajara	2002	2010	116	46
Human Carriage	Sibanor, Foni District	Dec-04	Apr-05	100	13
Monkey carriage	Mainly: Bijilo Forest Park and Abuko Nature Reserve,	Apr-11	May-11	82	31

Table lists the site where the patients were sampled or where the monkeys were trapped, the start and end dates of sampling, the number of S. aureus isolates that were characterised by MLST and the number of S. aureus genomes that were analysed in a given epidemiological class (i.e invasive disease, human and monkey carriage respectively).

Supplementary Figure 4. A google maps screen shot of the Western region of Gambia highlighting the sites where our isolates were sampled from in three previous studies.

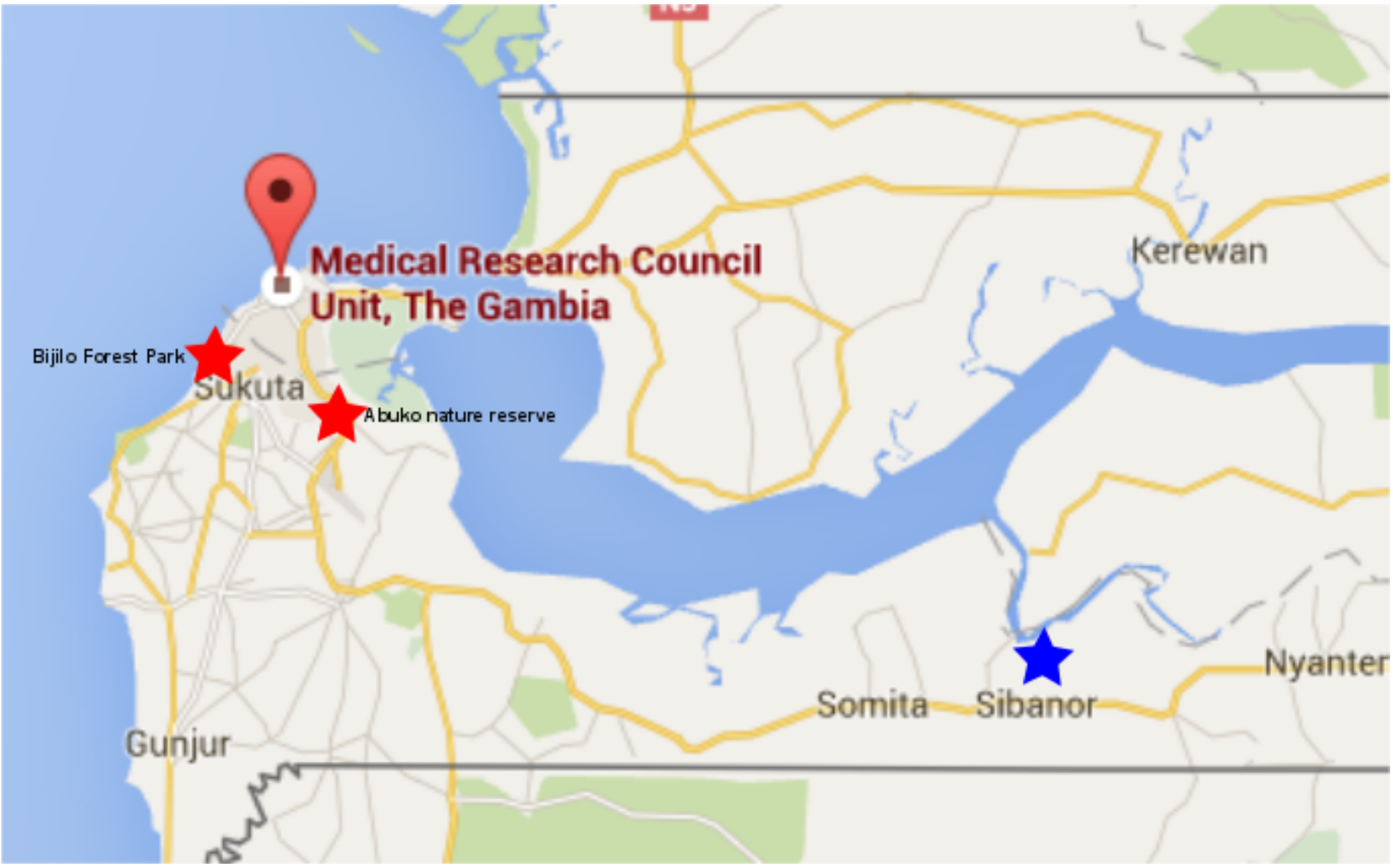

Red stars indicate the two sites, both within a 10 km radius from Fajara, where most of the monkey isolates were isolated from: Bijilo Forest Park and The Abuko Nature Reserve. The Blue star is showing Sibanor, a village in the Foni District (aproximately 80 km from Fajara), where the carriage study was carried out. The invasive disease isolates were collected from archived samples of patients that reported to the MRC clinic in Fajara.
